# Correlations Between Trimethylamine-N-Oxide, Megalin, Lysine and Markers of Tubular Damage in Chronic Kidney Disease

**DOI:** 10.3390/toxins17120592

**Published:** 2025-12-11

**Authors:** Stefania Kapetanaki, Samira Salihovic, Ashok Kumar Kumawat, Ziad A. Massy, Katarina Persson, Peter Barany, Peter Stenvinkel, Marie Evans, Isak Demirel

**Affiliations:** 1 School of Medical Sciences, Örebro University, Campus USÖ, 701 82 Örebro, Sweden; 2 Nephrology Department, Karolinska University Hospital, 141 86 Huddinge, Sweden; 3 Department of Nephrology, Ambroise Paré University Hospital, APHP, Boulogne-Billancourt, F-92100 Paris, France; 4 Inserm Unit 1018, Team 5, CESP, Hôpital Paul Brousse, Paris-Saclay University (UPS), Versailles Saint-Quentin-en-Yvelines University (UVSQ), F-94800 Villejuif, France; 5 Department of Clinical Science, Intervention and Technology, Division of Renal Medicine, Karolinska Institutet, 141 52 Stockholm, Sweden; peter.barany@ki.se (P.B.); marie.evans@ki.se (M.E.)

**Keywords:** TMAO, chronic kidney disease, megalin, lysine, albuminuria, tubular damage

## Abstract

Trimethylamine-N-oxide (TMAO), a gut microbiota-derived dietary metabolite, is linked to progression of chronic kidney disease (CKD). Megalin, a renal proximal tubule receptor crucial for albumin reabsorption, also plays a role in CKD. However, the relationship between them is not well explored. The aim of this study was to investigate if there are any correlations between the levels of TMAO, megalin, lysine and markers of tubular damage in CKD. Urinary metabolites (TMAO, choline, L-carnitine, betaine, lysine) and tubular markers (megalin, albumin, EGF, MCP-1) were quantified by LC-MS/MS and ELISA. Associations were evaluated using analysis of covariance (ANCOVA) adjusted for age and diabetes, with false discovery rate correction. Compared with controls, CKD patients showed higher urinary choline (FDR < 0.001), betaine (FDR = 0.007), lysine (FDR = 0.005), and soluble megalin (FDR < 0.001) but lower EGF and EGF/MCP-1 ratio (both FDR < 0.001). Correlation analyses revealed that serum TMAO was positively associated with soluble megalin and negatively with EGF/MCP-1 ratio. Choline, L-carnitine, and betaine were positively correlated with megalin. This cross-sectional study identifies associations between urinary metabolites, megalin, and tubular injury markers in advanced CKD. Although causality cannot be inferred, the results point to a potential metabolic–tubular link that should be explored in future longitudinal and mechanistic studies.

## 1. Introduction

Chronic kidney disease (CKD) is a complex disease related to several risk factors such as older age, obesity, hypertension, dyslipidemia, family history, and type 2 diabetes [1]. These risk factors trigger glomerular and tubulointerstitial damage which results in decreased glomerular filtration rate (GFR) and increased levels of albuminuria. CKD causes progressive kidney function decline and may progress to end-stage kidney disease (ESKD) in need of dialysis or kidney transplantation [1]. Approximately 13% of the world’s population suffer from CKD [2]. According to the World Health Organization (WHO), the global number of deaths due to CKD is 5–10 million annually [1]. The prevalence of CKD in the western world is rapidly rising due to increased aging and increased prevalence of CKD risk factors such as obesity, type 2 diabetes, hypertension and cardiovascular disease [1].

Trimethylamine-N-oxide (TMAO) is a uremic toxin, which is increased in CKD patients [3]. TMAO is generated from the oxidation of trimethylamine (TMA) which takes place in the liver and is catalyzed by the enzyme flavin monooxygenase 3 (FMO3). TMAO is eliminated mainly through urine in humans [4]. TMA is a product of gut microbiota metabolism of dietary organic compounds such as choline, betaine, L-carnitine, and phosphatidylcholine [4]. Eggs, red meat, fish, and dairy products are the main source of these compounds [5]. In the context of CKD, gut microbiota alteration leads to increased production of TMA and inflammation-mediated breakdown of enterocyte tight junctions. As a result, the supply of TMA to the liver through the circulation increases followed by increased TMAO production [6]. Altered gut microbiome and decreased glomerular filtration rate both contribute to the increased levels of TMAO in the plasma of CKD patients [3,7]. Increased plasma levels of TMAO are associated with increased all-cause mortality [8], increased risk for cardiovascular events [9,10], and increased atherosclerosis burden [11] in CKD patients [12,13].

Megalin and cubilin are two multiligand glycoproteins that form a receptor complex on tubular cells [14]. This complex reabsorbs albumin and a variety of low-molecular-weight proteins from the proximal tubular infiltrate [14,15]. Megalin is a large transmembrane protein belonging to the low-density lipoprotein (LDL) receptor family [14]. Cubilin is responsible for the binding of albumin, while megalin is essential for albumin internalization, which takes place in the tubular cell through the process of endocytosis [14,16]. The capacity of the megalin–cubilin complex is overwhelmed in the case of CKD due to increased albuminuria [14]. During CKD, there is a reduction in the expression of megalin and a reduction in tubular uptake of albumin [17,18], although the mechanism behind this is unclear. Furthermore, megalin can be shedded into the urine in a soluble form. There are two forms of soluble megalin in urine: a full-length form (C-megalin) and an ectodomain form (A-megalin) [15]. Urinary C-megalin is associated with microalbuminuria [19] and is increased in diabetes mellitus type 2 patients with diabetic nephropathy and increasing proteinuria [15]. Moreover, our previous study showed that there is an association between TMAO and megalin [20]. We found that TMAO downregulated megalin expression and albumin uptake in human proximal tubular cells in vitro [20].

Lysine is a known blocker of protein reabsorption in the proximal tubule [21]. L-lysine interferes with reabsorption of proteins by saturating the protein-binding sites on megalin [21,22]. Moreover, lysine was found to affect the cellular trafficking of the megalin–cubilin receptor complex [23]. Notably, lysine administration was proven to have a protective effect in hypertensive CKD [24]. Given the potential mechanistic link between lysine, megalin, and tubular protein reabsorption, we hypothesized that urinary lysine may be associated with megalin and markers of tubular dysfunction in CKD.

Despite the role of TMAO, megalin, and lysine in the proximal tubule, there are currently no observational studies that have investigated the association between them in CKD. The aim of this study was to investigate if there are any correlations between the levels of TMAO, megalin, lysine, and markers of tubular damage in CKD.

## 2. Results

### 2.1. Baseline Characteristics

Clinical and laboratory characteristics of healthy controls and CKD stage 4–5 patients are summarized in Table 1. The two groups were comparable with respect to gender distribution and serum calcium. However, patients with CKD were significantly older and had a higher BMI compared with controls. The prevalence of smoking and diabetes mellitus was also markedly higher in the CKD group. As expected, CKD patients had substantially lower eGFR and higher creatinine levels. They also exhibited significantly lower hemoglobin and albumin concentrations. In addition, serum phosphate and the prevalence of malnutrition (SGA < 5) were significantly higher in CKD patients. In contrast, total cholesterol levels were modestly lower in the CKD group.

### 2.2. TMAO, Choline, Betaine, and L-Carnitine Levels in CKD Patients and Healthy Controls

**Figure 1:**
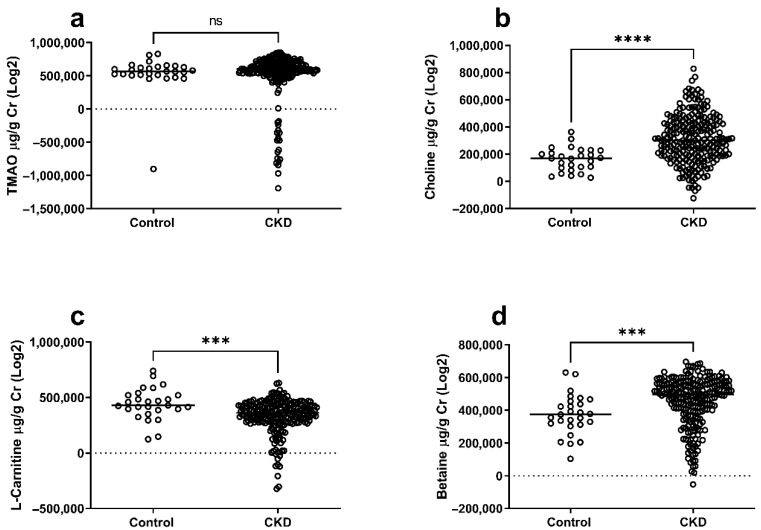
TMAO levels and its precursors in CKD patients compared to healthy controls. The levels of TMAO (**a**), choline (**b**), betaine (**c**), and L-carnitine (**d**) were determined in the urine of CKD patients compared to healthy controls. The horizontal line on the dot plot represents the median. Asterisks denote statistical significance (*** *p* < 0.001, **** *p* < 0.0001); ns, non-significance. Healthy control (*n* = 27), CKD patients (*n* = 233).

Urinary levels of TMAO and its precursors were first compared between CKD patients and healthy controls. In the unadjusted analysis, there was no significant difference in urinary TMAO between groups (Figure 1a). However, urinary choline (Figure 1b) and betaine (Figure 1d) were significantly higher, while L-carnitine (Figure 1c) was significantly lower in CKD patients compared with controls. As CKD patients were older and had a higher prevalence of diabetes, we then performed adjusted analyses using ANCOVA with age and diabetes as covariates. Even after adjustments, choline (β = 1.5 × 10^5^, FDR < 0.001) and betaine (β = 8.75 × 10^4^, FDR = 0.0067) remained significantly higher, while L-carnitine (β = −1.02 × 10^5^, FDR = 0.0024) remained significantly lower in CKD patients. Urinary TMAO remained not significantly different after adjustment (Table 2). With respect to covariate effects, age showed a modest negative association with urinary choline levels (*p* = 0.016), suggesting a potential decline in choline excretion with aging. Diabetes was independently associated with higher urinary choline (*p* = 0.0026), whereas no significant age or diabetes effects were observed for TMAO, betaine, or L-carnitine. These findings indicate that while age and diabetes influence choline metabolism, they do not fully explain the observed differences between CKD and control groups.

### 2.3. Soluble Megalin, Lysine, and Markers of Tubular Damage in CKD Patients and Healthy Controls

**Figure 2:**
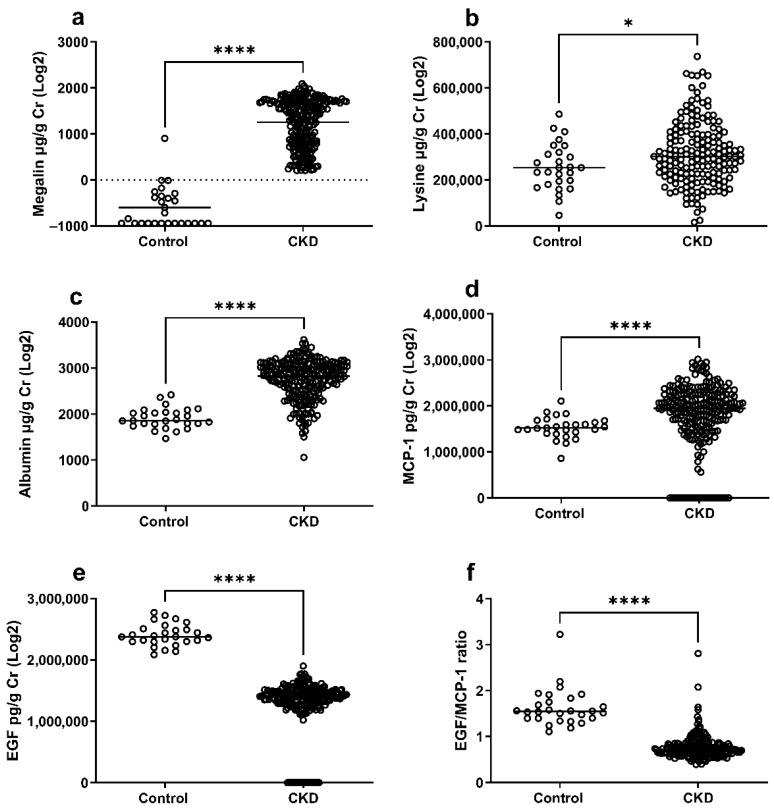
Soluble megalin, lysine, albumin, and markers of tubular damage in urine of CKD patients compared to healthy controls. The levels of soluble megalin (**a**), lysine (**b**), albumin (**c**), MCP-1 (**d**), EGF (**e**), and EGF/MCP-1 (**f**) were determined in the urine of CKD patients compared to controls. The horizontal line on the dot plot represents the median. Asterisks denote statistical significance (* *p* < 0.05, **** *p* < 0.0001); ns, non-significance. Healthy control (*n* = 27), CKD patients (*n* = 233).

Unadjusted analyses showed that soluble megalin (Figure 2a), lysine (Figure 2b), and albuminuria (Figure 2c) were all significantly elevated in CKD patients. Among tubular damage markers, urinary MCP-1 was increased (Figure 2d), while EGF (Figure 2e) and the EGF/MCP-1 ratio (Figure 2f) were significantly decreased in CKD patients. As CKD patients were older and had a higher prevalence of diabetes, we then performed adjusted analyses using ANCOVA with age and diabetes as covariates. In these adjusted analyses, megalin (β = 1.92 × 10^3^, FDR < 0.001), lysine (β = 8.81 × 10^4^, FDR = 0.0047), and albumin (β = 844, FDR < 0.001) remained significantly higher in CKD, whereas EGF (β = −1.1 × 10^6^, FDR < 0.001), and the EGF/MCP-1 ratio (β = −0.90, FDR < 0.001) remained significantly lower. However, MCP-1 was not significantly different between groups after adjustment (Table 2). Regarding covariate effects, age was negatively associated with megalin, lysine, and EGF levels, while diabetes was positively associated with EGF/MCP-1 ratio (*p* = 0.017) but not with megalin or lysine. These findings suggest that both aging and diabetes modestly influence tubular biomarker variability, yet the main differences between CKD and controls persist after adjustment.

### 2.4. Correlations Between TMAO, Megalin, Lysine, and Markers of Tubular Damage in CKD

**Figure 3:**
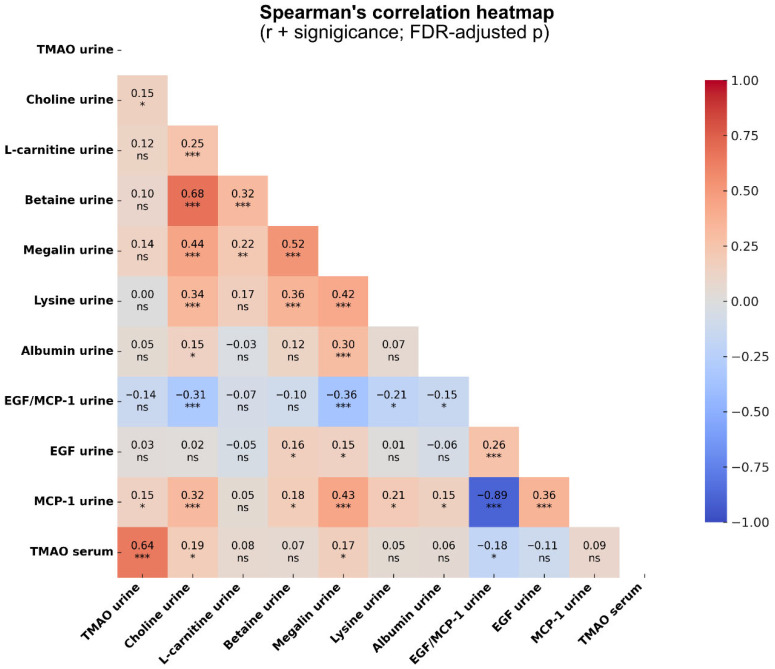
Correlation analysis between urine TMAO, choline, betaine, L-carnitine, megalin, lysine, albumin MCP-1, EGF, EGF/MCP-1 and serum TMAO. Each square represents Spearman’s correlation coefficient (r), with colors indicating correlation strength (red = positive, blue = negative). Numbers inside the squares are r-values, and statistical significance is indicated by FDR-adjusted *p*-values: ns (*p* ≥ 0.05), * (*p* < 0.05), ** (*p* < 0.01), and *** (*p* < 0.001).

Correlation analysis between urinary TMAO, its precursors, soluble megalin, lysine, albumin, and tubular damage markers is shown in Figure 3. After FDR correction, urine TMAO was positively associated with MCP-1. We also found that serum TMAO, previously measured in investigations based on the same cohort [25], remained positively associated with soluble megalin and negatively associated with urinary EGF/MCP-1 (Figure 3). Urine choline was also found to be positively associated with soluble megalin, lysine, albuminuria, and MCP-1 and negatively associated with the EGF/MCP-1 ratio (Figure 3). Urinary L-carnitine was positively associated with urine megalin, and urinary betaine was positively associated with soluble megalin, lysine, EGF and MCP-1. Furthermore, soluble megalin was positively associated with lysine and albuminuria and negatively associated with EGF/MCP-1. In addition, lysine and albuminuria were both positively associated with MCP-1 and negatively associated with EGF/MCP-1 (Figure 3).

## 3. Discussion

The current study investigates the correlations between TMAO, choline, L-carnitine, betaine, megalin, lysine, albuminuria, and markers of tubular damage in CKD patients. We found that urine levels of choline and betaine were increased, and L-carnitine was decreased in CKD patients compared to healthy controls. However, no difference was observed for TMAO in urine. ANCOVA adjustment for age and diabetes confirmed that these group differences were independent of major clinical covariates. TMAO and its precursors have all been linked to kidney dysfunction, through TMAO or independently [20,26,27,28,29]. In accordance, others have also found that plasma, but not urine levels of TMAO, were elevated in CKD patients compared to healthy controls [11]. These results underscore the central role of reduced glomerular filtration in TMAO accumulation. However, potential contributions from diet, gut microbiota composition, and tubular secretion cannot be excluded. Differences in urinary TMAO between groups may therefore have been masked by variations in nutritional status, which we partially accounted for using the Subjective Global Assessment (SGA), showing a higher prevalence of protein-energy wasting among CKD patients [30], suggesting that nutritional heterogeneity may also contribute to interindividual variability in urinary TMAO.

Next, we investigated the differences in soluble megalin levels in the urine of CKD patients. We have previously found that TMAO can modulate proximal tubular cells by decreasing the protein expression of megalin in vitro [20]. Others have also found that megalin expression is reduced during CKD [17]. In this study we found increased urinary levels of megalin in CKD patients. Urinary megalin has been found to be a marker of diabetic kidney disease progression [31]. However, the functional role of soluble urinary megalin is still unknown [31,32,33]. The elevated urinary megalin levels could result from increased shedding of tubular megalin associated with recycling processes or tubular damage. Furthermore, we also found that CKD patients had increased albuminuria, which is known to be associated with tubulointerstitial damage [34]. Megalin is responsible for the reabsorption of albumin, and urinary megalin has been shown to be associated with microalbuminuria [19]. Furthermore, the urinary lysine levels were found to be increased in CKD patients. This could also partly contribute to the increased albumin levels in urine, as lysine is known to bind to megalin and prevent protein reabsorption [21]. In our investigation, apart from albuminuria, we also used urine MCP-1, EGF, EGF/MCP-1 as markers of tubular damage. Urine EGF, MCP-1, and their ratio are considered indicators of rapid decline in kidney function and are used as markers of tubular damage and CKD progression [35,36,37,38]. The reduction in EGF and the EGF/MCP-1 ratio observed in CKD patients is consistent with more advanced tubular dysfunction, as previously described in chronic kidney disease. However, these markers are known to vary widely in CKD across different stages, comorbidities, and sampling conditions [35,36,37,38], and should therefore be interpreted as indicators of general tubular injury rather than as evidence of injury driven by any single mechanism. Although urinary MCP-1 was elevated in unadjusted analyses, this difference was not retained after adjustment for age and diabetes, suggesting that inflammatory comorbidities may partly account for its variability. However, it is important to note that the tubular damage could either cause, or result from, the increased urinary shedding of megalin and/or albuminuria. Covariate analyses further revealed that age was negatively associated with urinary choline, megalin, lysine, and EGF, whereas diabetes was independently associated with higher urinary choline and a higher EGF/MCP-1 ratio. These results suggest that while age- and diabetes-related processes modestly influence tubular biomarker variability, they do not fully explain the differences observed between CKD patients and controls.

To gain a deeper understanding of our results, we continued to evaluate the associations between TMAO, choline, L-carnitine, betaine, megalin, lysine, albumin, MCP-1, EGF, and EGF/MCP-1. We found that serum TMAO, but not urine TMAO, is positively correlated to urinary megalin and negatively correlated to EGF/MCP-1. This finding suggests that the high levels of serum TMAO in CKD patients are associated with tubular damage and enhanced megalin shedding. TMAO has been shown to induce tubular damage in rats [39]. The association between TMAO and soluble megalin, together with our in vitro findings showing that TMAO downregulates megalin expression in proximal tubular cells [20] and the reduced tissue megalin reported in CKD [17], supports a potential TMAO–megalin axis contributing to tubular dysfunction. These findings collectively support the observed decrease in albumin reabsorption during CKD. Looking at the correlation between megalin, lysine, EGF/MCP-1, and albumin, we found that they were all associated with each other, except for lysine and albumin. These findings further support the link between tubular damage, megalin, lysine, and albumin. Others have also found that albuminuria reflects tubular damage [34,40]. As megalin has been shown to be associated with microalbuminuria [19], and lysine is a megalin inhibitor [21], the association between lysine and albumin might have been observed at the early stages of CKD. Furthermore, we also found that urine choline, and betaine were positively associated with soluble megalin and lysine. Choline was also associated with albumin and EGF/MCP-1. The association of TMAO precursors, particularly choline, with megalin, lysine, albuminuria, and tubular damage further strengthen the connection between TMAO, its precursors, and CKD. Elevated levels of plasma choline have been suggested to be an indicator of tubular dysfunction and atherogenesis, and choline is also associated with kidney dysfunction in CKD [26]. High urinary choline and betaine likely reflect a combination of impaired tubular reabsorption and disturbed one-carbon metabolism, leading to osmolyte leakage and increased filtered load [41,42,43]. Betaine, which is osmolyte, is similarly lost during tubular stress and has been linked to renal osmolyte imbalance and disease progression [42]. In contrast, urinary L-carnitine was significantly decreased in CKD patients. This finding reflects impaired renal clearance, primarily due to the reduction in GFR, which reduces the filtered load, and altered tubular reabsorption via the OCTN2 transporter [44,45]. Others have also found that urinary choline levels were significantly associated with kidney dysfunction and poor prognosis. However, the observed link between urinary choline and kidney dysfunction may be related to the effects of TMAO [26]. In a murine model, TMAO and not choline was associated with renal function impairment [46]. Taken together, these findings highlight the intricate associations between TMAO and its precursors and megalin, lysine, and albumin, indicating that these interactions may play a significant role in tubular damage and the progression of CKD.

The key strength of this study lies in its novelty; it is the first to evaluate the in vivo relationships between TMAO, megalin, albuminuria, and tubular injury. The main limitation of the study is the relatively small number of healthy controls, which may affect the statistical power and the generalizability of the findings. The study was not prospectively powered; instead, we report effect sizes and 95% CIs and applied FDR control. A post hoc sensitivity analysis shows the design had ~80% power to detect moderate group effects (d ≈ 0.57; ≈0.52 with covariate adjustment), so smaller differences may have gone undetected. The relatively small control group further reduces precision and should be addressed in future, larger, balanced cohorts. While this sensitivity estimate provides a general indication of detectable effect sizes, it should be noted that effect-size estimates derived from markedly asymmetric cohorts (such as ours) tend to be less stable, and thus the true power may be more limited than suggested by the post hoc calculation. Moreover, CKD patients were not stratified by disease stage, which may have obscured stage-dependent trends in tubular and metabolic markers. In addition, detailed dietary data were unavailable, limiting our ability to account for nutritional variation known to influence TMAO and its precursors. Finally, the cross-sectional design precludes inference of causality, and longitudinal studies are needed to confirm the temporal relationships among TMAO, its precursors, and tubular injury.

In summary, the current study identified significant associations between TMAO and its precursors and soluble megalin, lysine, albuminuria, and markers of tubular injury in CKD. These findings provide new insight into potential metabolic–tubular interactions in CKD and form a basis for future mechanistic and longitudinal studies to clarify causality and therapeutic relevance.

## 4. Material and Methods

### 4.1. Patients and Study Design

Serum samples and 24 h urine samples from CKD stage 4–5 Swedish patients from the prospective cohort study European QUALity (EQUAL) were used in this study [47]. The samples were centrifuged within two hours of collection, aliquoted to avoid repeated freeze–thaw cycles, and stored at −80 °C until analysis. Ethical approval has been granted by the regional ethics review board in Stockholm, Sweden (Dr 2011/1821-31/4 and Dr 2024-02738-02). Written informed consent was obtained from all patients. A full description of the study has been published elsewhere [47]. Patients ≥ 65 years of age were included if their estimated glomerular filtration rate (eGFR) had decreased for the first time to ≤20 mL/min per 1.73 m^2^ during the previous 6 months (between March 2012 and February 2019). Patients were followed from inclusion until kidney transplantation, death, transfer to a non-participating center, withdrawal of consent, loss to follow-up, or the study’s end of follow-up, whichever occurred first.

Controls ≥ 60 years of age were randomly selected by the Statistics Bureau of Sweden (www.scb.se, accessed on 16 July 2025). The only exclusion criterion for selecting controls was a lack of willingness to participate in the study. The control population has been described previously [29]. The 24 h urine collection was approved by the regional ethics review board in Stockholm, Sweden (Dr 2016/1470-31). Written informed consent was obtained from all controls.

### 4.2. Data Collection

Circulating levels of albumin, creatinine, calcium, phosphatase, hemoglobin, and total cholesterol were analyzed according to certified methods at the Karolinska University Laboratory, Unit of Clinical Chemistry, Karolinska University Hospital, Sweden. Estimated glomerular filtration rate (eGFR) and measured GFR were evaluated as previously stated [29,47]. Data on pre-existing cardiovascular disease (CVD), including cerebrovascular disease, peripheral vascular disease, myocardial infarction, angina pectoris, congestive heart failure, left ventricular hypertrophy, and cardiac arrhythmias, were recorded. The 7-point subjective global assessment (SGA) tool was used to assess patients’ nutritional status, and SGA < 5 was defined as malnutrition.

### 4.3. Urine Liquid Chromatography–Tandem Mass Spectrometry

To investigate TMAO, choline, L-carnitine, betaine, lysine, and creatinine in urine, ultra-high pressure liquid chromatography–tandem mass spectrometry measurements were performed on an Acquity Premier UHPLC coupled to a Xevo TQ-XS triple quadrupole mass spectrometer (MS/MS) (Waters Corporation, Milford, MA, USA). Briefly, 10 µL of an internal standard mixture (including Creatinine-d3, Choline-d9, Carnitine-d3, TMAO-d9, Betaine-d11, and Tryptophan-d5) was added to 50 µL of urine. Protein precipitation was then achieved by adding 340 µL of ice-cold isopropanol–acetonitrile in a 1:2 ratio. The mixture was subsequently filtered using a Sirocco protein precipitation plate. A total of 2 µL of the urine sample extract was injected onto a 1.7 µm, 2.1 mm × 150 mm Acquity BEH AMIDE HILIC column, in combination with a guard column. For creatinine measurements, extracts were further diluted (1:1000) due to high concentrations and analyzed separately using the same method. The column temperature was 30 °C. The gradient mobile phases were A (20 mM ammonium formate in 100% Milli-Q and 0.1% formic acid) and B (0.1% formic acid in 100% acetonitrile). The flow rate was 0.3 mL/min, and the total run time per sample was 14 min. The analysis was performed using unispray ionization, operating in the positive ion mode. The source temperature was 150 °C, and the desolvation temperature was 350 °C. The cone gas flow was 150 L/h, and the desolvation gas flow was 700 L/h. Overall, the method was successfully validated in terms of the recovery and precision. The internal standard recoveries ranged from 62 to 107%. An external calibration curve (8 points, 75–1000 ng/mL) was constructed and showed R2 > 0.97–0.99 and 5.7–18.7% RSD. The LOD was defined as the average amount of trace of each analyte in the blank samples plus three standard deviations. The LOQ was defined as the average amount of the trace of each analyte in the blank samples plus five standard deviations. Serum TMAO data were obtained from previously published study based on the EQUAL cohort [25,47].

### 4.4. Measurement of Megalin, Albumin, EGF, and MCP-1 in Urine

Megalin, albumin, EGF, and MCP-1 levels in urine were analyzed by enzyme-linked immunosorbent assay (ELISA). Megalin was quantified using the human LRP2 ELISA kit (Abbexa, Cambridge, UK). Albumin was quantified using the human albumin kit (Duo set, ELISA, R&D Systems, Minneapolis, MN, USA). EGF was quantified using the human EGF kit (R&D Systems). MCP-1 was quantified using the human MCP-1 kit (R&D Systems). These assays are validated commercial kits. Each assay included a full calibration curve (R^2^ > 0.98), and intra- and inter-assay coefficients of variation were maintained below 10%. Internal quality control samples were analyzed on every plate to ensure assay precision and reproducibility.

### 4.5. Data Analysis

Urinary metabolite and protein concentrations were normalized to urinary creatinine to correct for variations in urine dilution. Data are expressed as mean ± SD or median (25th to 75th percentile) or percentage, as appropriate. Statistical significance was set at the level of *p* < 0.05. Mann–Whitney test was used to compare between two groups. Fisher’s exact test was used to evaluate categorical variables. Spearman’s correlation was used to determine correlations between variables.

To account for potential confounding, analysis of covariance (ANCOVA) was performed for each urinary biomarker, with CKD status as the main factor and age and diabetes as covariates. Regression coefficients (β) with 95% confidence intervals (CI) are reported. Because several biomarkers displayed non-normal distributions, model residuals were examined visually (Q–Q plots and standardized residuals versus fitted values) to evaluate normality and homoscedasticity, and no major deviations were observed. In parallel, generalized linear models (GLMs) with a gamma distribution and log link were fitted for biomarkers restricted to positive values to ensure robustness of the findings. A post hoc sensitivity analysis indicated that with 233 CKD and 27 control participants (α = 0.05), the study had approximately 80% power to detect moderate group effects (Cohen’s d ≈ 0.57; ≈0.52 when accounting for covariates in ANCOVA). Multiple testing was controlled using the Benjamini–Hochberg false discovery rate (FDR) method, with FDR-adjusted *p* < 0.05 considered statistically significant. No data were excluded; inspection of standardized residuals and leverage statistics confirmed that no extreme outliers disproportionately influenced the regression estimates. All analyses were performed in jamovi v2.5 and GraphPad Prism v9.

## Figures and Tables

**Table 1:** Demographic and laboratory characteristics of controls and CKD patients.

	Controls (*n* = 27)	CKD 4–5 (*n* = 233)	*p* Value
**Age, years**	70 ± 5	75 ± 6	**0.0002**
**Male gender, *n* (%)**	22 (81)	160 (69)	0.191
**BMI, kg/m^2^**	25.3 ± 3.1	27.6 ± 5	**0.032**
**Smoking, *n* (%)**	7 (26)	16 (7)	**0.0046**
**Diabetes, *n* (%)**	2 (1)	88 (38)	**0.0011**
**CVD, *n* (%)**	2 (1)	38 (16)	0.395
**eGFR MDRD**	77 (72–95)	20 (17–23)	**0.0001**
**Diastolic BP, mmHg**	NA	76 (70–85)	
**Systolic BP, mmHg**	NA	148 (133–161)	
**SGA overall assessment**	7 (7–7)	6 (5–7)	**0.0001**
**SGA < 5 (malnourished), *n* (%)**	0 (0)	27 (12)	0.0888
**α-Blocker, *n* (%)**	NA	4 (1.7)	
**β-Blocker, *n* (%)**	NA	69 (30)	
**ACEi/ARB, *n* (%)**	NA	96 (41.4)	
**Lipid-lowering, *n* (%)**	NA	78 (33.6)	
**Diuretic, *n* (%)**	NA	106 (45.7)	
**Creatinine, µmol/L**	83 (70–91)	271 (225–311)	**0.0001**
**Albumin, g/L**	38 (36–41)	36 (33–38)	**0.0011**
**Calcium, mmol/L**	2.3 (2.2–2.4)	2.28 (2.19–2.37)	0.82
**Phosphate, mmol/L**	1 (0.9–1.1)	1.3 (1.1–1.5)	**0.0001**
**Hemoglobin, g/L**	144 (133–149)	118 (108–127)	**0.0001**
**Total cholesterol, mmol/L**	4.7 (4.1–5.8)	4.6 (3.8–5.5)	**0.024**

Abbreviations and definitions: BMI, body mass index; CVD, including cerebrovascular disease, peripheral vascular disease, myocardial infarction, angina pectoris, congestive heart failure, left ventricular hypertrophy, and cardiac arrhythmias; eGFR, estimated glomerular filtration rate; ACEi/ARB, angiotensin-converting enzyme inhibitor/angiotensin receptor blocker; SGA, subjective global assessment, assess patients’ nutritional status, SGA < 5 was defined as malnutrition; BP, blood pressure. Values are expressed as number (%), mean ± SD or median (25th–75th percentile) as appropriate. NA: not available. Statistical significance is defined by *p* < 0.05.

**Table 2:** Association of urine TMAO, choline, betaine, L-carnitine, megalin, lysine, albumin, MCP-1, EGF, and the EGF/MCP-1 ratio with CKD status, age, and diabetes.

	CKD vs. Control β (95% CI); FDR	Age Effect β (95% CI); FDR	Diabetes Effect β (95% CI); FDR
**TMAO urine**	807 (−1.46 × 10^5^ to 1.48 × 10^5^); FDR = 0.991	−4.1 × 10^3^ (−1.09 × 10^4^ to 2.71 × 10^3^); *p* = 0.237	2.3 × 10^4^ (−6.91 × 10^4^ to 1.15 × 10^5^); *p* = 0.624
**Choline urine**	1.5 × 10^5^ (8.07 × 10^4^ to 2.2 × 10^5^); FDR < 0.001	−3.95 × 10^3^ (−7.17 × 10^3^ to −731); *p* = 0.016	6.71 × 10^4^ (2.35 × 10^4^ to 1.11 × 10^5^); *p* = 0.0026
**L-carnitine urine**	−1.02 × 10^5^ (−1.65 × 10^5^ to −3.98 × 10^4^); FDR = 0.0024	−2.55 × 10^3^ (−5.44 × 10^3^ to 332); *p* = 0.0826	2.44 × 10^4^ (−1.47 × 10^4^ to 6.34 × 10^4^); *p* = 0.22
**Betaine urine**	8.75 × 10^4^ (2.61 × 10^4^ to 1.49 × 10^5^); FDR = 0.0067	−2.69 × 10^3^ (−5.52 × 10^3^ to 149); *p* = 0.0632	2.74 × 10^4^ (−1.1 × 10^4^ to 6.58 × 10^4^); *p* = 0.161
**Megalin urine**	1.92 × 10^3^ (1.71 × 10^3^ to 2.14 × 10^3^); FDR < 0.001	−10.4 (−20.4 to −0.466); *p* = 0.040	−51.7 (−186 to 82.9); *p* = 0.45
**Lysine urine**	8.81 × 10^4^ (2.73 × 10^4^ to 1.49 × 10^5^); FDR = 0.0047	−3.16 × 10^3^ (−5.97 × 10^3^ to −347); *p* = 0.0278	2.53 × 10^4^ (−1.27 × 10^4^ to 6.33 × 10^4^); *p* = 0.191
**MCP-1 urine**	2.58 × 10^5^ (−3.32 × 10^4^ to 5.5 × 10^5^); FDR = 0.0914	−2.14 × 10^3^ (−1.56 × 10^4^ to 1.13 × 10^4^); *p* = 0.754	2.45 × 10^4^ (−1.58 × 10^5^ to 2.07 × 10^5^); *p* = 0.791
**EGF urine**	−1.10 × 10^6^ (−1.27 × 10^6^ to −9.26 × 10^5^); FDR < 0.001	1.83 × 10^3^ (−6.19 × 10^3^ to 9.85 × 10^3^); *p* = 0.654	−8.36 × 10^4^ (−1.92 × 10^5^ to 2.49 × 10^4^); *p* = 0.13
**EGF/MCP-1**	−0.902 (−1.05 to −0.758); FDR < 0.001	−2.54 × 10^−5^ (−0.00667 to 0.00662); *p* = 0.994	−0.11 (−0.199 to −0.0196); *p* = 0.017
**Albumin**	844 (667 to 1.02 × 10^3^); FDR < 0.001	−4.21 (−12.4 to 3.98); *p* = 0.312	28.9 (−81.9 to 140); *p* = 0.608

Abbreviations and definitions: Values represent regression coefficients (β) with 95% confidence intervals (CI) derived from ANCOVA models. *p*-values are reported for age and diabetes effects, and Benjamini–Hochberg false discovery rate (FDR) adjusted *p*-values are reported for CKD vs. control comparisons. Statistically significant results at adjusted *p* < 0.05.

## Data Availability

The data presented in this study are available on request from the corresponding authors due to the privacy of individuals who participated in the study.
